# Natural Language Processing in Clinical Research Recruitment: A Scoping Review Enriched with Stakeholder Insights

**DOI:** 10.1002/eahr.60014

**Published:** 2025-09-27

**Authors:** Lara Bernasconi, Georg Avakyan, Frédérique Hovaguimian, Regina Grossmann

**Affiliations:** ^1^ Doctoral student in biomedical ethics and law at the University of Zurich and University Hospital Zurich; ^2^ Master's student at the Federal Institute of Technology Zurich; ^3^ Scientific associate in data research services at University Hospital Zurich; ^4^ Director of the Clinical Trials Center at University Hospital Zurich

**Keywords:** clinical research, patient recruitment, patient autonomy, equity, anonymization, consent, natural language processing (NLP), artificial intelligence (AI), AI ethics

## Abstract

We conducted a scoping review to characterize natural language processing (NLP) applications in clinical trials recruitment and conducted semistructured interviews to obtain stakeholders’ perspectives on these technologies, with a focus on ethical considerations. The scoping review focused on English‐language original articles published from January 2021 to June 2024, sourced from Ovid Medline. Data extracted included the characteristics of NLP systems, their evaluations, and ethical considerations regarding patient autonomy and equity. Additionally, semistructured interviews with experts from various specialties were conducted, and the data were analyzed using thematic analysis. Most of the 47 eligible articles focused on NLP models for electronic health records screening. The literature predominantly emphasized the models’ accuracy and efficiency, while ethical considerations received little attention. Interview findings underscored the need for more ethical reflection and real‐world implementation analysis, revealing differing opinions on anonymization, consent, and the impact of NLP tools on fair opportunities. NLP applications for participant recruitment in clinical research are in early stages, with a gap between ethical discourse and reporting in current literature. Practical guidelines are needed for implementing and reporting ethical aspects throughout the lifecycle of NLP applications, along with empirical research to assess their ethical impact.

While there has been substantial progress in conducting clinical research over the past decades, it remains complex and resource intensive. To ensure the success of future clinical research, the integration of emerging technologies and a collective effort to improve existing models and processes is crucial.^1^ Various artificial intelligence (AI) applications have the potential to streamline the planning, execution, and analysis of clinical trials, offering the possibility of transforming the future of the field.^2^ One area that could benefit from AI is the recruitment of study participants. Among the various challenges in clinical research, recruitment stands out as a significant hurdle.^3^ This process includes evaluating eligibility through interviews, physical exams, and a thorough review of electronic health records (EHRs) to make informed decisions based on patient data. Failing to recruit and retain the necessary participants can significantly jeopardize both the internal and external validity of a research study.^4^

Natural language processing (NLP) is a subfield of AI and involves computers analyzing and processing human language with the use of various algorithms, tools, and methods. Models are trained on large datasets from various sources, including open‐source resources, EHRs, and biomedical ontologies. Data are converted into numerical forms the model can understand, and the model learns by making predictions and adjusting its parameters to reduce errors. NLP encompasses both natural language understanding and natural language generation, with diverse applications across fields like machine translation, text categorization, information extraction, summarization, and dialogue systems.^5^ All these NLP applications can be employed to support the recruitment process in clinical research. For instance, NLP can analyze vast datasets, including EHRs and social media, to identify suitable participants for clinical trials. It can predict patient responses, target specific demographics, and enhance participant matching by focusing on individuals most likely to benefit from a treatment.^6^ Regarding natural language generation, NLP‐powered chatbots can support the screening process, providing trial information and allowing participants to ask questions.^7^ Additionally, generative adversarial networks (GANs) have recently been used in NLP to create highly accurate synthetic data from relatively small amounts of real‐world information.^8^ This synthetic data may offer an alternative to real patient data and could be seen as a way to overcome recruitment challenges.

While several studies have explored this area, there is little literature evaluating the use of NLP applications in clinical research recruitment. For instance, although Ghim et al. examined the role of large language models in clinical trials,^9^ their research did not specifically focus on recruitment applications. Ismail et al. investigated how AI technologies support recruitment in clinical research,^10^ but their study was a narrative review that did not involve a systematic literature search and was not limited to specific AI technologies. Finally, Idnay et al. conducted a systematic review on NLP but concentrated specifically on its use for eligibility prescreening.^11^ Moreover, the ethical dimension is underrepresented in these reviews. While NLP technologies hold significant promise, we believe that it is crucial to consider their potential ethical implications. Susser et al. and Shanley et al., for instance, have highlighted concerns about synthetic data, including persistent privacy challenges, issues of accuracy, and risks of biases.^12^

Our study aimed to characterize and assess recent evidence on NLP applications in clinical research recruitment, with a particular focus on ethical aspects. Extensive guidance exists on designing AI technologies that incorporate moral values.^13^ The World Health Organization (WHO) guidelines, for example, emphasize key ethical principles for AI in health care.^14^ In our study, we explored whether the current literature reflects the implementation of these WHO guidelines, particularly concerning autonomy and equity. Additionally, we captured stakeholders’ perspectives on the challenges and opportunities of these technologies, providing insights into the practical implications of these ethical principles for NLP‐driven recruitment in clinical research.

## STUDY METHODS

We conducted a mixed‐method study combining findings from a scoping review and semistructured interviews with experts from different specialties. The study methods and results are reported according to the preferred reporting items for systematic reviews and meta‐analyses extension for scoping reviews (PRISMA‐ScR) checklist and the consolidated criteria for reporting qualitative research (COREQ).^15^ Since this study did not collect any personal or sensitive data from interviewees for the analysis, it falls outside the scope of the Swiss Human Research Act and therefore did not require ethics approval. Informed consent was obtained from all interviewees.

Eligibility criteria for the scoping review were established before the database search. No review protocol was published. We included original articles in English, published from January 2021 onward, that described the development or implementation of NLP systems for participant recruitment in clinical research. Recruitment in clinical research was defined as identifying, selecting, and enrolling participants in systematic studies to evaluate new treatments, understand disease mechanisms, and improve health outcomes. We considered NLP as a branch of AI and linguistics that enables computers to understand and generate human language.^16^ We applied no restrictions on disease domain, nor on type and phase of clinical research. However, we excluded reports on AI applications unrelated to recruitment support, as well as studies focused on preclinical research or public health. The search strategy (see appendix 1, which is available online; information about accessing this material is in the “Supporting Information” section at the end of this article), designed for the Ovid Medline database, used a combination of medical subject heading terms and keywords related to NLP (artificial intelligence, large language model, natural language processing, synthetic data) and clinical research (clinical research, clinical studies, clinical trials, medical research). The last search was performed on June 30, 2024. Two authors (LB and GA) independently conducted the initial title and abstract screening, resolving any discrepancies by consensus. Full‐text screening and data extraction were performed by the same authors using a data collection form specifically designed for this review. To ensure consistency, LB and GA independently reviewed a sample of five articles, resolving discrepancies and refining the extraction tool. The remaining articles were divided equally between the two researchers for full‐text screening and data extraction. As a quality control measure, five other articles were double‐checked. The extracted data included details such as author, title, publication year, geographic location of the authors, disease area, characteristics and development stage of the NLP systems, systems evaluations, and ethical considerations (see appendix 1). We restricted our analysis to two ethical principles: autonomy, which requires meaningful human oversight, protection of privacy, and ensuring informed consent; and equity, which calls for inclusiveness and being mindful of potential biases and their impact on individuals and society.^17^ Data were either classified according to predefined categories in the extraction tool or relevant text passages were copied into the template. The results were then summarized descriptively.

In a second step, one‐hour interviews were conducted in English via teleconference in June and July 2024. An interview guide was developed iteratively by the authors. Questions were formulated to gather opinions on the use of NLP to support recruitment in clinical research (see appendix 2 online). Emphasis was placed on the ethical aspects, especially concerning autonomy and equity, as was done in the literature review. The guide was pilot tested with a layperson to ensure comprehensibility before implementation. Purposive sampling was employed to recruit a data engineer (Interviewee 1), a data protection officer (Interviewee 2), a representative of the ethics committees (Interviewee 3), an expert in patients’ involvement and rights (Interviewee 4), a clinical researcher (Interviewee 5), and an AI ethicist (Interviewee 6). Candidates were approached via email. One did not respond, and one participant suggested by a candidate declined to participate. The participants, comprising three females and three males, were all familiar with the field of clinical research, though their level of expertise in AI and ethics varied. Five interviewees work in Switzerland, and one in Germany. LB conducted all interviews and audio‐recorded the discussions with the participants’ permission. An overview of the thematic fields and a description of the use cases identified from the scoping review were provided to the interviewees in advance. The audio files were transcribed using Amazon Transcribe. LB applied exploratory thematic analysis to

**We advocate for the development of clear guidelines on implementing and reporting ethical considerations in studies involving NLP solutions for participant recruitment.**

the data,^18^ combining inductive and deductive qualitative analysis. The authors developed an agreement on categorizing the codes. Appendix 2 includes examples of quotations along with their coding and categorization. The interview participants were given the opportunity to review and provide feedback on the manuscript and appendix 2.

## SCOPING REVIEW

### Characteristics of articles

A total of 799 articles were identified through the database search. After removing duplicates, 507 articles were screened by title and abstract, with 63 retained for full‐text review. Following review, 44 articles met the inclusion criteria. Additionally, three articles were included from the screening of references in review articles. We ultimately included 47 articles in our final dataset (figure 1 online).^19^ A summary of the included studies is provided in appendix 1.

The literature on NLP‐driven recruitment in clinical research showed a modest upward trend, with a publishing rate of 1.0 article/month during 2021‐2022 and 1.3 articles/month during 2023‐June 2024. Most of the studies focused on the development and training validation of AI systems (n = 37), while a small number reported on real‐world applications (n = 7). Three articles presented an AI technology without details on its technical development or implementation. Of the 47 studies, 34 were conducted in the United States, four in China, and two in France. The remaining studies (n = 7) included one study from Canada, and others from various European and Asian countries. None of the reviewed studies were conducted in Africa, Australia, or South America. Most studies involved models trained on English data sources, with only two algorithms trained on Chinese and two on French data sets.

Apart from the studies that investigated multiple research areas (n = 19), oncology was the dominant field (n = 13), followed by cardiology (n = 5), neurology (n = 3), and immunology (n = 2). The remaining studies (n = 5) investigated other clinical areas.

More than half of the studies (n = 25) reported on NLP systems that are open source. In two cases, the technology was explicitly noted as non‐open source, while the availability of the tools and models was not specified for the rest (n = 20).

### NLP use cases

Six NLP applications to support recruitment in clinical research were identified from the reviewed articles and are represented in figure 2 (online). Most articles (n = 28) were assigned to the category “patient screening from EHR.” Most of these (n = 20) focused on identifying potential participants for specific studies. In a few cases (n = 3), a patient‐centric approach focusing on understanding and prioritizing the patient's specific needs and medical history was used to find the most suitable study for a selected patient. Four studies combined both approaches (bidirectional matching). Finally, one article aimed at predicting the screening success of potential study participants through EHR screening (see figure 2).

The second most prominent use case was “parsing of eligibility criteria” (n = 14), which was further divided into three subcategories: creating cohort queries (n = 8), structuring and coding eligibility criteria (n = 5), and creating a queries database (n = 1). Parsing of eligibility criteria involves the process of extracting, interpreting, and structuring the often complex and detailed criteria used to determine a patient's suitability for a clinical trial and is a building block for EHR screening. Indeed, one study investigated both use cases simultaneously and was therefore assigned to both categories.

The creation of synthetic data for clinical research was explored in two studies, as was NLP‐based social media analysis. One social media study aimed to classify users and identify patient cohorts, while the other conducted sentiment analysis to improve the recruitment strategy. The prediction of enrollment rates and the use of screening chatbots were the least frequently investigated use cases, with only one article identified for each category. The screening chatbot was used to determine if patients qualified for the study through online conversations. Predicting enrollment rates was the only identified use case that relied on characteristics available before the study began and did not require patient data for implementation.

### Models’ assessment and ethical considerations

**Figure 3:**
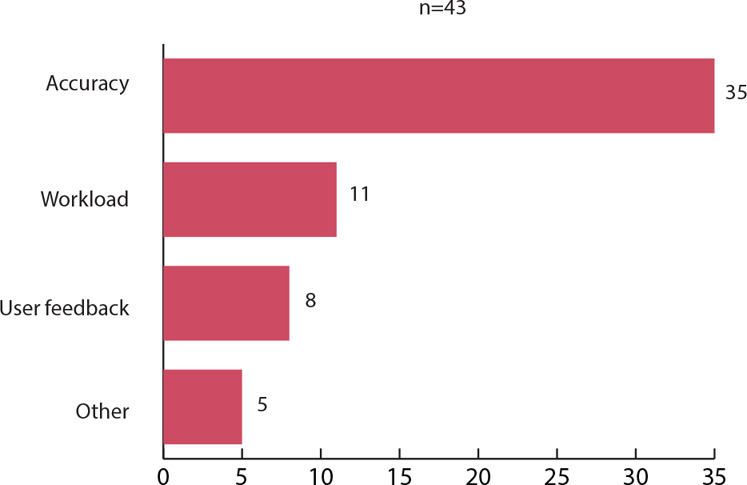
Evaluation of NLP Technologies Articles were assigned to all applicable evaluation categories. Types of evaluations that were conducted only in one or two articles are summarized under the category “other.” Four articles did not evaluate the technologies presented and were excluded from the graph.

The NLP systems were empirically evaluated in 43 of the reviewed studies, each assessing one or more aspects (see figure 3). Evidence regarding the accuracy of the investigated technology was provided in 35 articles. The impact of AI on the workload of clinical researchers was analyzed in 11 studies, while users’ feedback was assessed in eight studies. In one study, the research team analyzed and quantified the generalizability of the results generated by the NLP system, while another examined the amount of required human intervention. The two studies on synthetic data analyzed the quality of the generated data, with one also examining privacy preservability. The screening chatbot was evaluated for its effectiveness in obtaining consent.

Regarding the level of automation and the role of human oversight, 27 articles provided relevant statements, describing the range of human intervention from minimal to moderate or highlighting collaborative efforts. The remaining articles (n = 20) did not address this topic.

**Figure 4:**
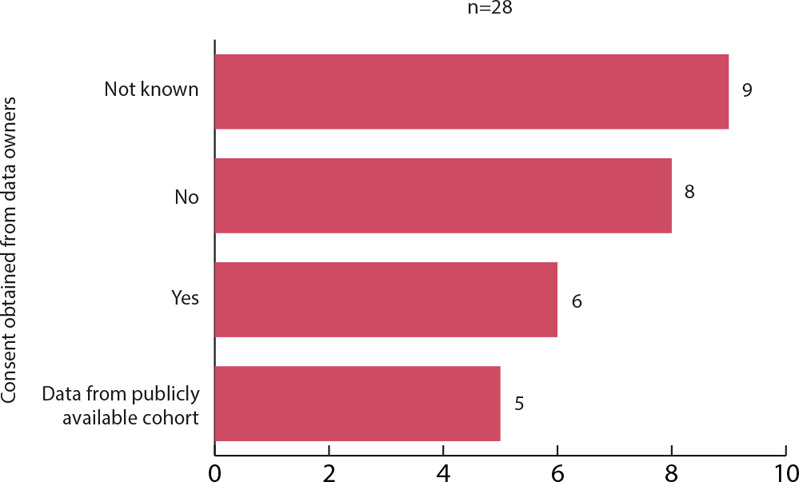
Data Owner Information about Data Involvement in the Research Project Only articles involving patient data were included in the graph. Studies that used data from publicly available cohorts have been categorized separately because patient consent is managed by the database creators, not by the researchers of the reviewed articles.

Of the 28 studies that involved patient data to train the models, six explicitly declared that consent was obtained from the data owners. Waivers of consent were obtained for eight studies because the data were deidentified or reused in the context of other studies. No information about consent was provided in nine articles, of which three specified the use of deidentified data. Five studies used data from publicly available deidentified cohorts (see figure 4).

Comments related to biases (gender, age, ethnicity) and the impact of AI tools on equity were found in five articles. Of these, three articles addressed the limitations of the AI systems, while two highlighted a positive effect on the equity of clinical research. Quantitative data analysis supported the statements in only two cases.

## STAKEHOLDER PERSPECTIVES

### Contextual factors and human elements of recruitment

Figure 5 (online) summarizes the key themes that emerged from the interview data. Several interviewees emphasized that when discussing NLP in patient recruitment for clinical research, it is important to consider contextual aspects, which are themselves shaped by evolving individual and societal values. These contextual aspects include the current AI hype, which influences how people perceive and react to these technologies, as well as characteristics of clinical research. Interview participants described clinical research as inherently associated with ethical and operational challenges, such as ensuring equal access, maintaining compliance, and mitigating biases.

Additionally, many interviewees highlighted the importance of human factors in patient recruitment. One interviewee elaborated on this theme, saying: “The recruitment is a multi‐layered topic. There are exclusion criteria that are defined by the protocol, that is the easier part. The more difficult part is really to ensure that the patient is participating as a partner in that research project. That depends more on the psychology aspects, the attitude of the patient altogether. His characteristics and his personality is impacted by the disease in a way that nobody really explores, neither as part of the diagnostic, nor especially as part of the enrollment process. The behavior and the reactions of the investigator also have a massive impact on the ultimate reaction of the patient” (Interviewee 4, expert in patients’ involvement and rights). Importantly, interviewees not only emphasized the many dimensions underlying the decision process of a study participant, but also the necessity of human interaction, intuition, and experience, particularly with specific patient populations. 

### Opportunities of NLP‐driven clinical research recruitment

Within this framework, NLP appears to hold promise for improving the recruitment process. Interviewees expressed hopes for enhanced efficiency and speed in recruitment. Interviewee 3 (representative of the ethics committees) emphasized the potential for better quality (more reliable, complete, and valid data) by recruiting patients who are potentially more compliant. As noted by Interviewee 4, AI may also bring a degree of standardization that improves the predictability and reliability of recruitment, eliminating geographical limitations. Another aspect highlighted by several interviewees is the potential for a more patient‐centered approach. They believe AI could help adapt and explain a study dynamically, accounting for individual needs and differences. This would significantly enhance informed and autonomous decision‐making for patients.

### Challenges of NLP‐driven clinical research recruitment

Despite the opportunities, interviewees also mentioned various challenges. Regarding the development of NLP models, challenges primarily revolved around the limited availability and low quality of training data, which can lead to biased and underperforming models. Additionally, data availability is inevitably tied to data protection issues. For the deployment phase, issues such as model interoperability and the limitations of current models’ performance were highlighted, impacting trust in these tools. Trust issues were not solely linked to model performance. Some interviewees noted that certain use cases could jeopardize the already delicate patient‐researcher relationship. As one interviewee explained: “During a normal screening process there is a contact between the study team and the potential study participants. If you automate the process, this is not there anymore. Everything is done behind the back of participants. And this is an ethical problem. It breaks the trust, which is important for the informed consent process and decision of the patient whether to participate in the trial or not” (Interviewee 3, representative of the ethics committees).

Additionally, ethical concerns were raised about the potential misuse of tools, such as coercing participation in studies via chatbots or creating profiles or assessments of individuals based on their activities, interactions, and behaviors on social media platforms and other online environments. Interviewee 6 (an AI ethicist), commented on these issues: “Regarding the analysis of social media, I think that the game is not worth the candle, it's too much reputational risk for, I would say, insufficient biomedical gain. I mean, social media platforms might contain some signals, but I think the quality of the data is insufficient. The number of inferences that you have to make and the amount of data that you need to train accurate predictions is too big. Imagine that you are a clinical center that is recruiting for a specific trial on a sexually transmitted disease, which is usually an ethically controversial topic. How would a patient feel about being selected for a study based on inferences made on his or her habits? People might feel violated, people might feel exposed.” The quality control of the tools also faces challenges, particularly due to the specificity of the different models, which makes validating each application more laborious.

### Topics with ambivalent value

Besides clear challenges and opportunities, the analysis of the interview data highlighted a dualistic tension for some topics, including in‐house solutions, anonymization of training data, open sourcing, and impact on equity. According to some interviewees, in‐house solutions provide stronger data protection and customization, but these solutions are more difficult and costly to implement. They may also hinder centralization, which can facilitate the development of more reliable models and offer patients greater control over their data usage. While anonymization of training data enhances data protection and fosters innovation, it may not always provide the desired level of privacy and can sometimes be misused to circumvent consent requirements, at the cost of transparency. Interviewees expressed differing opinions on these issues, with some also questioning whether broad consent forms are sufficient for this type of research and whether current consent texts adequately cover the use of data for training AI models. One interviewee noted: “When patients sign the general consent, what we are discussing here is probably not what they have in mind. I'm just not sure whether the general consent is really adequate for this kind of use of patient data” (Interviewee 2, data protection officer).

Open sourcing can enhance resource sharing but may also increase the risk of misuse, complicate data protection‐related issues, and may raise concerns over incentives and intellectual property. Of note, some interviewees recognized the potential for AI to foster fair opportunities. Said one interviewee: “You [the researcher] are probably more fair, I think really fair because you treat everybody the same way. AI does not have, you know, like certain biases or sympathies towards a person” (Interviewee 5, clinical researcher).

In contrast, others expressed concerns about the risk of introducing biases and inequities if not carefully managed. As previously mentioned, social profiling may arise in the context of social media analysis. Interviewee 6 (AI ethicist) also highlighted a philosophical dilemma concerning the creation of synthetic data: if synthetic data are designed to accurately replicate the datasets they are based on, they will inevitably reflect the demographic biases embedded within the original data. This raises an essential question: what constitutes a “good” synthetic dataset? Should it faithfully mirror the original data, preserving its inherent limitations, or should it be designed to correct for these limitations to provide a more equitable representation?

### Stakeholder suggestions

Interviewees provided various suggestions for how to tackle the many dimensions underlying NLP‐driven clinical research recruitment. First, to improve the development of NLP tools for recruitment in clinical research, it appears crucial to address biases and conduct thorough risk assessments. As one interviewee pointed out, “Many tools just focus on performance and not on the risk. What we lack as of now is risk assessment. There are very few methods on how to really assess and quantify the risk. And actually all the decisions around these tools should be risk‐based” (Interviewee 1, data engineer). “Risk‐based” refers to making decisions by explicitly considering the potential harms, vulnerabilities, or unintended consequences that a tool might introduce.

Second, deployment should be transparent and consider the broader social context, maintaining human involvement to avoid overly passive participant roles and fostering trust. An AI ethicist (Interviewee 6) raised this issue, saying: “When it comes to assessing the use of technology in the medical sphere, from an ethical point of view, it's never just about looking at the features of the technology itself. What we implement in the clinic is not only the technology, it is a social system. Around the technology, there are actors that operate and maintain it, there are users and subjects. It's really a complex social ensemble that we implement.”

Third, regular human supervision and monitoring were highlighted as essential complements to ensure quality; and fourth, at a broader level, interviewees suggested establishing a robust regulatory and ethical framework grounded in societal moral consensus and concrete quality standards. This framework should address all phases of the tool's lifecycle. Finally, ongoing involvement and training of both the general public and the research community should be prioritized.

## DISCUSSION

Our study revealed that research on NLP applications in clinical research recruitment appears to be in its early stages, with most articles focusing on tool development rather than real‐world implementation. A clear emphasis emerged on NLP technologies for screening EHRs and parsing eligibility criteria to facilitate this task. While accuracy and efficiency were commonly evaluated, ethical considerations received less attention. Stakeholder interviews highlighted the need for more in‐depth ethical reflection and practical implementation analysis. The interview findings also revealed that the stakeholders in our study had differing views on anonymization and consent requirements for NLP training data, and the potential impact of NLP tools on equity.

### A lot of efficiency and too little ethics

The finding that research has particularly concentrated on EHR screening aligns with previous research on the application of AI in clinical trials.^20^ With a longer history of exploration, more standardized processes likely exist for EHR screening. In contrast, the other use cases may suffer more from the lack of standards mentioned in the interviews. Another interpretation may be that EHR screening better meets the apparent focus on performance and efficiency. Although the interviewees highlighted that NLP holds promise for enhancing quality and patient‐centeredness, most papers did not prioritize these aspects. In terms of system evaluations, the emphasis in the literature was largely on accuracy, with some consideration given to reducing the workload of clinical researchers. However, implementation aspects such as user feedback were less frequently addressed, and the literature seems to lack evidence regarding the implementation of WHO guidelines, offering minimal attention to ethically relevant factors. In our study, we focused particularly on the principle of equity, especially in relation to biases and inclusiveness; and autonomy, which encompasses privacy, informed consent, and human oversight.^21^

### Inadequate addressing of biases

The interviewees identified limited availability and poor quality of training data as key challenges, potentially leading to biased and underperforming models. In discussing synthetic data, for example, Interviewee 6 (AI ethicist) raised a critical question: what constitutes a “good” synthetic dataset? Should it faithfully replicate the original data, including its inherent limitations, or should it be adjusted to overcome these limitations and offer a more equitable representation? Concerns about biases have been previously expressed for AI applications in clinical trials.^22^ However, in this study we found that the risk of biases is addressed in only a few articles. Interviewee 1 highlighted the lack of validated methods for assessing and quantifying risks, which likely contributes to these deficiencies. As noted by Piorkowski et al., current AI risk assessments are mostly qualitative, but suitable metrics for quantitative assessments are emerging. Moving toward these metrics could lead to more objective and comparable evaluations.^23^

### Unclear impact on inclusiveness

Conflicting opinions from interviewees emerged regarding the impact of AI tools on inclusiveness and fair opportunities. While NLP can risk perpetuating inequities, it may also promote fairer participant identification. Weissler et al. similarly highlighted this dual potential, adding that while AI could reinforce inequities, it might also help mitigate them by monitoring and drawing attention to biases in clinical research.^24^

### A risk‐based approach for privacy and consent

Regarding privacy and consent, the reviewed literature lacked sufficient details to assess the quality of anonymization and the adequacy of patient information and consent. This complicates the assessment of compliance with privacy regulations and ethical standards. The interviews revealed divergent opinions on anonymization and consent requirements for NLP training data, as well as on the adequacy of current regulations and consent practices, especially in relation to broad consents, underscoring the need for further discussion involving all stakeholders. In this context, risk‐assessment emerged as a crucial element to drive decisions.

### Need for human oversight

Human oversight was considered crucial by the interviewees, especially at this stage of technological development. In contrast to that, in the literature, human oversight was discussed in less than two‐thirds of the articles. Guidelines for “meaningful human control” should be tailored specifically to this context.^25^

### Uncertainties in real‐world implementation

More broadly, uncertainty persists regarding the real‐world implementation of these tools. Existing AI implementation frameworks have been identified as inadequate.^26^ Ismail and colleagues have emphasized that future research should focus on incorporating AI systems in real clinical trial recruitment processes to assess their efficacy.^27^ Our interviews further indicate that an early and comprehensive analysis of the entire implementation context is crucial for advancing the field. This includes not only technical factors but also the importance of human elements in the recruitment process, as well as ethical considerations regarding privacy and equity, and also trust. The interviewees raised concerns particularly regarding the implementation of EHR screening, social media analysis, and screening chatbots. For EHR screening, the most researched use case, the concerns particularly focused on the lack of transparency toward patients and the risk of eroding their trust. The authors of this paper share this concern, noting that it could have serious negative implications for the field. Clinical research cannot afford further trust issues, as skepticism and fear are already major barriers to patient participation in trials.^28^

### Study limitations

This study has some limitations to consider. First, we restricted our review to English‐language publications, though the risk of overlooking significant NLP articles was considered minimal as English is the predominant language in scientific research. Second, as a scoping review, we did not assess the quality of the evidence; the aim was to provide an overview of existing applications. A further limitation of this study was the small number of stakeholder interviews conducted, which may limit the generalizability of the statements. To mitigate this effect, however, we included a variety of interviewees with diverse professional backgrounds, expertise, and perspectives on NLP. This selection of key experts allowed for in‐depth insights into the topic under investigation, thereby ensuring that the findings are well‐rounded and relevant. Third, although no patient was interviewed, the perspectives of patients may have been partially represented by the experts in bioethics or those working directly with patients. Finally, while the manual coding of themes introduced some subjectivity, the authors developed an agreement on categorizing the codes. Despite its limitations, we believe this study offers a comprehensive overview of NLP applications for clinical research recruitment, with a particular focus on specific ethical aspects. By incorporating perspectives from various stakeholders, our analysis provides practical insights into the challenges and opportunities of integrating these technologies, grounded in real‐world experiences.

## CONCLUSION

NLP applications for recruitment in clinical research are still in the early stages of development, and our study highlights a significant gap between the ethical discourse and the existing literature on these applications. While published articles mainly emphasize accuracy and efficiency, they often overlook ethical considerations regarding the development and the implementation of the proposed models. To address this, we advocate for the development of clear guidelines on implementing and reporting ethical considerations in studies involving NLP solutions for participant recruitment. These guidelines should cover the practical application of these tools, ensuring that ethical assessments encompass the entire lifecycle of the technology. As a first step, multidisciplinary expert panels should review current legal and ethical frameworks to reach a moral consensus on controversial topics such as anonymization and consent requirements for training data. The interview data suggest that incorporating a risk‐based approach could be advantageous in this process. Empirical evaluation and discussion are needed to understand the implications for privacy, equity, and trust.

## ACKNOWLEDGMENTS

We sincerely thank Amanda Ramirez Ramos, Christa Stamm, Pietro Gervasoni, Ingrid Klingmann, Adrian Egli, and Alessandro Blasimme for participating in the interviews and providing their personal perspective on the topic. We acknowledge the use of ChatGPT 3.5 to enhance the manuscript text and correct sentences. No content was generated entirely by the chatbot. Prompts included requests for improvements, reformulations, and simplifications.

## Supporting information

Appendices 1 and 2, and figures 1, 2, and 5 are available in the “Supporting Information” section for the online version of this article and via *Ethics & Human Research*'s “Supporting Information” page: https://www.thehastingscenter.org/supporting-information‐ehr/.

Supporting information

Supporting information

Supporting information

Supporting information

Supporting information
