# Supplementary material for: Natural Language Processing in Clinical Research Recruitment: A Scoping Review Enriched with Stakeholder Insights
Source: Ethics Hum Res. 2025 Sep 27;47(5):13–23. doi: 10.1002/eahr.60014 (PMC12476210; doi:10.1002/eahr.60014)
Supplement: Supplementary file 1 — Supporting information [file EAHR-47-13-s002.pdf]

Appendix 1

Natural Language Processing in Clinical Research Recruitment: *A Scoping Review Enriched with Stakeholder Insights*

LARA BERNASCONI, GEORG AVAKYAN, FRÉDÉRIQUE HOVAGUIMIAN, AND REGINA GROSSMANN

Search strategy in Ovid Medline

First Search run on 14. February 2024

	Search	2021 - 13.02.2024
1	exp Artificial Intelligence as Topic /	73896
2	(LLM$ or (Large$ adj Language$ adj Model$) or NLP$ or (natural$ adj language$ adj processing$)) .mp.	7619
3	(synthetic$ adj3 data$).mp.	2966
4	exp Clinical Studies as Topic/	31781
5	((clinical$ adj trial$) or (clinical$ adj stud$) or (clinical$ adj research$) or (medical$ adj research$)).mp.	189888
6	1 and 2 and 3	10
7	1 and 2 and 4	25
8	1 and 3 and 4	2
9	1 and 3 and 5	13
10	1 and 2 and 5	171
11	2 and 4	35
12	2 and 5	375
13	2 and 4 and 3	0
14	2 and 5 and 3	0
	Sum	631

[mp=title, book title, abstract, original title, name of substance word, subject heading word, floating sub-heading word, keyword heading word, organism supplementary concept word, protocol supplementary concept word, rare disease supplementary concept word, unique identifier, synonyms, population supplementary concept word, anatomy supplementary concept word]

Second Search Run on 30. June 2024

	Search	14.02.2024 – 29.06.2024
1	exp Artificial Intelligence/	8759
2	(LLM$ or (Large$ adj Language$ adj Model$) or NLP$ or (natural$ adj language$ adj processing$)) .mp.	1913
3	(synthetic$ adj3 data$).mp.	454
4	exp Clinical Studies as Topic/	4217
5	((clinical$ adj trial$) or (clinical$ adj stud$) or (clinical$ adj research$) or (medical$ adj research$)).mp.	23493
6	1 and 2 and 3	2
7	1 and 2 and 4	4
8	1 and 3 and 4	0
9	1 and 3 and 5	2
10	1 and 2 and 5	39
11	2 and 4	5
12	2 and 5	113
13	2 and 4 and 3	0
14	2 and 5 and 3	3
	Sum	168

Inclusion criteria

- Original articles on NLP applications for recruitment in clinical research
- No restrictions on disease domain, population demographics (e.g., age, sex), and type/phase of clinical research the NLP system is applied to

Exclusion criteria

- Other applications than recruitment
    i. AI-powered medical interventions
    ii. AI as outcome generator (e.g. research on big data for biomarker discovery)
    iii. Focus on other phases of biomedical research (e.g. drug discovery)
    iv. Focus on other healthcare areas (e.g. public health)
    v. Focus on other medical research management applications
- Full text not accessible
- English text not available
- Reviews. However, we checked the bibliography of the identified reviews to ensure that all relevant studies were identified.

Summary of included studies and extracted data

**Paper and Research Team**					**Study**		**Tool**				**Tool evaluation**				**Ethics**		
Title	*Accession number*	*First Author*	*Year*	Country *(Institution first author)*	*Disease Area*	*Data Source language*	*Area of application - Category*	*Area of application -sub-category*	*Open source*	*Development vs real-world application*	Accuracy	Workload	User Feedback	Other	*Pat. information and anonymiz.*	*Human supervision discussed?*	Equity and bias reflection
Improving the Efficiency of Clinical Trial Recruitment Using an Ensemble Machine Learning to Assist With Eligibility Screening	34296815	T. Cai	2021	USA	Immunology	English	screening from EHR	subject identification	NK	Training Validation	Y	Y	N	N	NK	Y	N
Can synthetic data be a proxy for real clinical trial data? A validation study	33863713	Z. Azizi	2021	Canada	Oncology	English	synthetic data		NK	Training Validation	N	N	N	Y-quality synthetic data set	NK - anonymous data	N	N
Participatory Design of a Clinical Trial Eligibility Criteria Simplification Method	34042820	Y. Fang	2021	USA	Cardiology	English	parsing of eligibility criteria	creation of cohort queries	Y	Training Validation	N	N	Y	N	NK	Y	N
Clinical Inflection Point Detection on the Basis of EHR Data to Identify Clinical Trial-Ready Patients With Cancer	34097438	K. L. Kehl	2021	USA	Oncology	English	screening from EHR	patient-centric trial recommendation	NK	Training Validation	Y	N	N	N	N	N	Y
A Comparison between Human and NLP-based Annotation of Clinical Trial Eligibility Criteria Text Using The OMOP Common Data Model	34457154	X. Li	2021	USA	Mixed	English	parsing of eligibility criteria	creation of cohort queries	Y	Training Validation	Y	N	N	N	NA- no patients involved	N	N
A knowledge base of clinical trial eligibility criteria	33813032	H. Liu	2021	USA	Mixed	English	parsing of eligibility criteria	create a database of criteria	Y	NA	Y	N	N	N	NA- no patients involved	N	N
Analysis of Population Differences in Digital Conversations About Cancer Clinical Trials: Advanced Data Mining and Extraction Study	34554099	E. A. Perez	2021	USA	Oncology	English	analysis of social media	sentiment analysis to improve recruitment strategy	NK	real-world application	NA	NA	NA	NA	NK - anonymous data	Y	Y
Identifying Patient Phenotype Cohorts Using Prehospital Electronic Health Record Data	33315497	R. Stemerman	2021	USA	Cardiology	English	screening from EHR	subject identification	Y	Training Validation	Y	Y	N	N	NK	Y	Y
Transformer-Based Named Entity Recognition for Parsing Clinical Trial Eligibility Criteria	34414397	S. Tian	2021	USA	Mixed	English	parsing of eligibility criteria	structure/code eligibility criteria	Y	Training Validation	Y	N	N	N	NA- no patients involved	Y	N

Accuracy of an artificial intelligence system for cancer clinical trial eligibility screening: retrospective pilot study	33769304	T. Haddad	2021	USA	Oncology	English	screening from EHR	subject identification	NK	Training Validation	Y	N	N	N	N	N	N
Semantic categorization of Chinese eligibility criteria in clinical trials using machine learning methods	33858409	H. Zong	2021	China	Mixed	Chinese	parsing of eligibility criteria	structure/code eligibility criteria	NK	Training Validation	Y	N	N	N	NA- no patients involved	N	N
Batch enrollment for an artificial intelligence-guided intervention to lower neurologic events in patients with undiagnosed atrial fibrillation: rationale and design of a digital clinical trial	34033803	X. Yao	2021	USA	Cardiology	English	screening from EHR	subject identification	NK	real-world application	NA	NA	NA	NA	Y	Y	Y
APERITIF - Automatic Patient Recruiting for Clinical Trials Based on HL7 FHIR	34042705	A. Banach	2021	Germany	Mixed	English	screening from EHR	subject identification	N	Training Validation	Y	N	N	N	Data from publicly available cohort	N	N
Natural Language Processing for Patient Selection in Phase I or II Oncology Clinical Trials	34197179	J. Delorme	2021	France	Oncology	French	screening from EHR	prediction of successful screening /dose-limiting toxicity period	Y	Training Validation	Y	N	N	N	Y	Y	N
Recruitment in a research study via chatbot versus telephone outreach: a randomized trial at a minority-serving institution	34741513	Y. J. Kim	2021	USA	Immunology	English	screening chatbot	-	Y	real-world application	N	N	N	Y- effectivity in obtaining consent	Y	N	Y
Prediction of clinical trial enrollment rates	35202402	C. Bieganek	2022	USA	Mixed	English	prediction of enrollment rates		NK	Training Validation	Y	N	N	N	NA- no patients involved	N	N
Combining human and machine intelligence for clinical trial eligibility querying	35426943	Y. Fang	2022	USA	Mixed	English	parsing of eligibility criteria	creation of cohort queries	Y	Training Validation	Y	N	Y	Y- required human intervention	NA- no patients involved	Y	N
Implementation of Machine Learning Pipelines for Clinical Practice: Development and Validation Study	36525289	L. J. Kanbar	2022	USA	Pediatric Emergency	English	screening from EHR	subject identification	NK	real-world application	Y	Y	Y	N	NK	Y	N

Parsable Clinical Trial Eligibility Criteria Representation Using Natural Language Processing	37128426	J. Kim	2022	USA	Oncology	English	parsing of eligibility criteria	creation of cohort queries	Y	Training Validation	Y	N	N	N	NA- no patients involved	N	N
A comparative study of pre-trained language models for named entity recognition in clinical trial eligibility criteria from multiple corpora	36068551	J. Li	2022	USA	Mixed	English	parsing of eligibility criteria	creation of cohort queries	Y	Training Validation	Y	N	N	N	NA- no patients involved	N	N
Evaluation of Criteria2Query: Towards Augmented Intelligence for Cohort Identification	35673021	C. Liu	2022	USA	Mixed	English	parsing of eligibility criteria	creation of cohort queries	Y	Training Validation	N	N	Y	N	NA- no patients involved	Y	N
A Unified Machine Reading Comprehension Framework for Cohort Selection	34236972	Y. Xiong	2022	China	Mixed	English	screening from EHR	subject identification	NK	Training Validation	Y	N	N	N	Data from publicly available cohort	N	N
Discovering monogenic patients with a confirmed molecular diagnosis in millions of clinical notes with MonoMiner	35976265	D. W. Wu	2022	USA	Monogenic Diseases	English	screening from EHR	subject identification	Y	Training Validation	Y	N	N	N	Data from publicly available cohort	N	N
Identifying Patients With Inflammatory Bowel Disease on Twitter and Learning From Their Personal Experience: Retrospective Cohort Study	35917151	M. Stemmer	2022	Israel	Gastroenterology	English	analysis of social media	users classification	Y	real-world application	Y	N	N	N	N (retrospective consent for 3 examples containing direct quotes)	N	N
Effective matching of patients to clinical trials using entity extraction and neural re-ranking	37451494	W. Kusa	2023	Austria	Mixed	English	screening from EHR	bi-directional matching	Y	Training Validation	Y	N	N	N	Data from publicly available cohort	Y	N
OncoCTMiner: streamlining precision oncology trial matching via molecular profile analysis	37935585	Q. Xu	2023	China	Oncology	English	screening from EHR	bi-directional matching	Y	NA	NA	NA	NA	NA	NA- no patients involved	Y	N
LeafAI: query generator for clinical cohort discovery rivaling a human programmer	37550244	N. J. Dobbins	2023	USA	Mixed	English	parsing of eligibility criteria + screening from EHR	creation of cohort queries+ subject identification	NK	Training Validation	Y	N	N	N	NK	Y	N
Clinical research staff perceptions on a natural language processing-driven tool for eligibility prescreening: An iterative usability assessment	36638583	B. Idnay	2023	USA	Neurology	English	screening from EHR	subject identification	Y	Training Validation	N	N	Y	N	NA- no patients involved	Y	N

Matching Patients to Clinical Trials with Large Language Models	37576126	Q. Jin	2023	USA	Mixed	English	screening from EHR	subject identification	Y	Training Validation	Y	Y	N	N	Data from publicly available cohort	N	N
Automated Matching of Patients to Clinical Trials: A Patient-Centric Natural Language Processing Approach for Pediatric Leukemia	37428994	S. Kaskovich	2023	USA	Oncology	English	screening from EHR	patient-centric trial recommendation	Y	Training Validation	Y	Y	N	N	NA- no patients involved	Y	N
Prescreening in oncology trials using medical records. Natural language processing applied on lung cancer multidisciplinary team meeting reports	36964666	F. Jacobs	2023	France	Oncology	French	screening from EHR	subject identification	NK	Training Validation	Y	N	N	N	N	N	N
Piloting an automated clinical trial eligibility surveillance and provider alert system based on artificial intelligence and standard data models	37041475	S. Meystre	2023	USA	Mixed	English	screening from EHR	subject identification	NK	real-world application	Y	N	N	N	N	Y	N
Named Entity Recognition and Normalization for Alzheimer's Disease Eligibility Criteria	38283164	Z. Sun	2023	USA	Neurology	English	parsing of eligibility criteria	structure/code eligibility criteria	NK	Training Validation	Y	N	N	N	NA- no patients involved	N	N
Patient Phenotyping for Atopic Dermatitis with Transformers and Machine Learning	37693571	A. Wang	2023	USA	Allergology	English	screening from EHR	subject identification	NK	Training Validation	Y	N	N	N	NK - anonymous data	Y	N
Synthetic Data Generation by Artificial Intelligence to Accelerate Research and Precision Medicine in Hematology	37390377	S. D'Amico	2023	Italy	Hematology	English	synthetic data		Y	Training Validation	N	N	N	Y-quality synthetic data set	Y	N	N
Text Classification of Cancer Clinical Trial Eligibility Criteria	38222417	Y. Yang	2023	USA	Oncology	English	parsing of eligibility criteria	structure/code eligibility criteria	NK	Training Validation	Y	N	N	N	NA- no patients involved	Y	N
Large Language Models for Healthcare Data Augmentation: An Example on Patient-Trial Matching	38222339	J. Yuan	2023	USA	Cardiology	English	screening from EHR	bi-directional matching	NK	Training Validation	Y	N	N	Y-generalisability of results	NK	N	N
Automatic assessment of patient eligibility by utilizing NLP and rule-based analysis	38082656	P. P. Tun	2023	Singapore	Cardiology	English	screening from EHR	subject identification	N	Training Validation	Y	N	N	N	Y	Y	N
Digital ECMT Cancer Trial Matching Tool: an Open Source Research Application to Support Oncologists in the Identification of Precision Medicine Clinical Trials	36657092	P. O'Regan	2023	UK	Oncology	English	screening from EHR	patient-centric trial recommendation	Y	real-world application	NA	NA	NA	NA	Y	Y	N

Evaluation of an artificial intelligence-based clinical trial matching system in Chinese patients with hepatocellular carcinoma: a retrospective study	38388861	K. Wang	2024	China	Oncology	Chinese	screening from EHR	subject identification	NK	Training Validation	Y	Y	N	N	N	Y	N
AutoCriteria: a generalizable clinical trial eligibility criteria extraction system powered by large language models	37952206	S. Datta	2024	USA	Mixed	English	parsing of eligibility criteria	structure/ code eligibility criteria	Y	Training Validation	Y	N	N	N	NA- no patients involved	Y	N
Matching Patients to Accelerate Clinical Trials (MPACT): Enabling Technology for Oncology Clinical Trial Workflow	38269982	N. V. Do	2024	USA	Oncology	English	screening from EHR	bi-directional matching	NK	NA	N	Y- Qualitative feedback from users	N	N	NA- no patients involved	Y	N
Utilizing Large Language Models for Enhanced Clinical Trial Matching: A Study on Automation in Patient Screening	38854210	J. Beattie	2024	USA	Mixed	English	screening from EHR	subject identification	NK	Training Validation	Y	Y	Y	N	N	Y	N
Sociotechnical feasibility of natural language processing-driven tools in clinical trial eligibility prescreening for Alzheimer's disease and related dementias	38447587	B. Idnay	2024	USA	Neurology	English	screening from EHR	subject identification	Y	Training Validation	N	N	Y	N	N	Y	N
Distilling large language models for matching patients to clinical trials	38641416	M. Nievas	2024	USA	Mixed	English	screening from EHR	subject identification	Y	Training Validation	Y	Y	N	N	NA- no patients involved	Y	N
Criteria2Query 3.0: Leveraging generative large language models for clinical trial eligibility query generation	38697494	J. Park	2024	USA	Mixed	English	parsing of eligibility criteria	creation of cohort queries	Y	Training Validation	Y	Y	Y	N	NA- no patients involved	Y	N
Automating Clinical Trial Matches Via Natural Language Processing of Synthetic Electronic Health Records and Clinical Trial Eligibility Criteria	38827083	Victor M. Murcia	2024	USA	Mixed	English	screening from EHR	subject identification	Y	Training Validation	Y	Y	N	N	NA- no patients involved	N	N
