# Supplementary material for: Natural Language Processing in Clinical Research Recruitment: A Scoping Review Enriched with Stakeholder Insights
Source: Ethics Hum Res. 2025 Sep 27;47(5):13–23. doi: 10.1002/eahr.60014 (PMC12476210; doi:10.1002/eahr.60014)
Supplement: Supplementary file 2 — Supporting information [file EAHR-47-13-s005.pdf]

Natural Language Processing in Clinical Research Recruitment: *A Scoping Review Enriched with Stakeholder Insights*

Lara Bernasconi, Georg Avakyan, Frédérique Hovaguimian, and Regina Grossmann

Appendix 2

Interview guide

Thematic Field 1: General opportunities and challenges

1. What benefits do you see in integrating AI into medical research recruitment?
2. What challenges do you see in integrating AI into medical research recruitment?
    i. You may want to consider different perspectives: patients, researchers, AI developers, regulatory bodies?
3. Do you think that the benefits outweigh the challenges, or is it rather the other way around?

Thematic Field 2: Tools development and regulation of employment

4. In your opinion, when should patients be informed that their data is used to train such AI tools?
    i. Even if the data is de-identified?
5. How should the AI tools implementation be regulated?
    i. Who gets to decide when the use of the AI tools is justified?
    ii. Independent certification required? By whom?
    iii. Should the AI tools be open for everyone to use?
6. Who should play a leading role in the development and implementation of AI tools in this field—industry, hospitals, universities, government? Why?

Thematic Field 3: Use cases

7. What is your gut reactions to the use cases?
    i. Which one do you find most / less problematic and why?
    ii. Do you have any safety and/or privacy concerns?
8. What level of human-supervision do you wish for these tools?
9. In your opinion, in which cases should patients be informed about the use of the AI tool in a study?

Thematic Field 4: Outlook

10. How do you think these tools may affect the
    I. role of patients in medical research (e.g. more or less power?)?
    II. attitude of patients toward medical research?
    III. relationship between study patients and researchers?
11. How do you think these tools might impact patients' access to medical research?
12. Can you think of other AI applications that could support recruitment in medical research?
13. Are there any issues related to patient recruitment in medical research that AI tools cannot address?

Is there anything else you would like to add?

use-cases:

a) Use AI to automatically identify potential research participants from electronic medical histories or match patients to the most suitable studies.
b) Use AI chatbots to determine if people qualify for medical studies through online conversations (can include analysis of emotions or not)
c) Use AI to analyse social media posts to identify potential participants for medical research, classify users, or improve recruitment strategies.
d) Use AI to create synthetic patients for simulations/predictions: fake data that mimics real patient data but were created based on real-world information.
e) Use AI to predict enrolment rates for medical studies based on characteristics available before the study begins (no patients/patients data involved).

Examples of quotations and coding tree

Theme	Subtheme	Code	Example excerpts
Context	Social context	• AI hype	• At the moment AI has a very big hype, you know, everybody talks about it, everybody is talking about ChatGPT et cetera and that generates a lot of expectations. I think by doing research in this field, I'm just a bit more sceptical because I realize what works and what does not work. Medicine is really complex because humans are very complex and diseases are complex. And this is why I think this will also apply to the field of medical studies, this complex and we really need to invest into this. I also see that there is huge potential behind it, but there needs to be investment. (I5)
	Intrinsic challenges of clinical research	• Issue with compliance<br>• Risk of bias in clinical research	• Compliance is an issue in clinical trials. The advantage of the AI tool is also that it can help you identify compliant patients. There is a risk of bias, of course. We need to identify it and address it. Also without AI clinical trials may be biased, for example due to selective dropouts. E.g., a patient that experiences side effects decides to withdrew from the trial without giving any justification for his/her decision (I3)
		• Equal access as general problem	• Equal access in clinical research is actually a general problem independent from AI. (I1)<br>• I think in general, participation in clinical research is perhaps skewed towards the more educated people because they can better assess what is the need of a study, et cetera. (I5)
	Human factors	• Human individuality	• The recruitment is a multi-layered topic. There are exclusion criteria that are defined by the protocol, that is the easier part. The more difficult part is really to ensure that the patient is participating as a partner in that research project. That depends more on the psychology aspects, the attitude of the patient altogether, his characteristics and his personality is impacted by the disease in a way that nobody really explores, neither as part of the diagnostic, nor especially as part of the enrolment process. The behaviour and the reactions of the investigator also have a massive impact on the ultimate reaction of the patient. (I4)<br>• Whether to participate in clinical research is a very delicate personal choice and it very much depends on how a patient understands the trade-offs of benefits and burdens in participation. So, of course, people that have um let's say less clinical options are more eager to participate. And so are people for whom scientific research has some value independently of their personal condition. (I6)<br>• The investigator - patient relationship has already a trust problem because patients become in the first instance suspicious when they get offered a clinical trial. Depending also on the type of disease. Patients with life threatening diseases and rare diseases are more open to research. But in let's say broad therapeutic areas where there are a lot of treatment alternatives, the proposal to participate in a clinical trial can kill the trust between the patient and the doctor. (I4)
		• Importance of human interaction	• AI cannot substitute the relationship between investigator and patient. This relationship is essential, especially for the informed consent process. (I3)<br>• Participation of the patient totally depends on the human to human interaction. How well do you explain something to a human being because there are levels of empathy with it. (I5)
		• Importance of human intuition and experience	• When you are recruiting for special populations, rare disorders, I think that in those cases, it's more a matter of good old clinical judgment...it's a much more nuanced choice around enrolment. (I6)<br>• I'm doubtful that the AI can be trained in a way to answer study specific questions where it's just an experience of a physician. (I5)
		• AI cannot affect human psychology	• A patient may say yes on day one and then the next day the patient comes back to the doctor and says, no, I've thought about I, I do not want to participate. Or the effect of a first exposure to the trial situation, that is also not predictable. We lose patients when they have gone through the first assessments and then they say, no, I don't want to have that hassle again and I don't want to participate anymore. (I4)

	Context changeability	• Recognizing dynamism	• I think ethics is a discussion about, you know, what the society wants, et cetera. The perception can change. Studies which were OK 10 years ago, they would probably be nowadays be way more sceptical despite the fact it's the same law, you know what I mean? You see this very much currently in the discussion about data protection. How important data protection is as an example because obviously new technologies come in, people are more aware, they read more about it. So the fulfilment you need to have are also changing. And the ethics committees are also reacting differently. (I5)<br>• It is a developing field. What I say you is my current opinion on the subject but if we conduct this interview in six months, I may have a different opinion. (I3)
		• Inconsistent behaviour regarding data protection	• People are completely inconsequent. On one hand, they are very much fighting for protection of their personal data everywhere and are very much upset when they get approached with advertisements, but on the other hand, they put the names of the babies on the car, post things on social media,... (I4)
Opportunities	Improve the recruitment process	• Improve efficiency and speed of recruitment	• Recruitment is a limiting factor for clinical studies in general. It's really increasingly complicated to enrol patients. And also the more let's say sophisticated to study, the more difficult it is to find patients. Of course, AI could automate the analysis of available data to match prospective participants to research studies. And this could happen also in a semi-automated way, meaning it could happen in the background, without involving too much um human labour. (I6)<br>• With AI you can speed up the identification of the potential patient to be then later contacted and included in a trial. You increase performance, increase speed, decrease cost of course, because it is automated you don't have someone that has to go through all the patient charts or check out of patients forums on the internet and so on and so forth. (I3)
		• Improve quality of recruitment	• I think it would increase consistency and the quality of the study. (I5)<br>• The advantage of the AI tool is also that it can help you identify compliant patients. (I3)<br>• use case b: To the extent that these chat bots are proficient in reading emotions, it could even be better than the status quo of today. I mean, we are not aware of this very subtle psychological aspects or care during recruitment processes today. So this could even improve things. (I6)
		• Improve predictability and reliability in recruitment	• It would be very important that we improve recruitment under all circumstances and make it more predictable and, and more reliable. We have big deficit on that in Europe, we have all this fragmentation into different countries. We have borders which limit the recruitment options. So there is definitely a need to find better and more reliable tools to improve recruitment and to predict recruitment. AI certainly holds promises for this area. (I4)
		• Patient-centred approach	• I think AI could help to better configure the different e-consent elements for particular patient groups either from an age perspective or from a male-female perspective or from a disease perspective or stage of disease. I think AI could really help to sophisticate that and to make that more adapted to the personalities that the needs of these different patients. (I4)<br>• I think AI could help adapt and explain a study in a dynamic way. At the moment, I think most of the AI models are simply text based interactions. So the emotional sensing as an example is based on how does the patient reply, how fast does a patient write. I think having multimodal AI would be better. (I5)
Challenges	Obstacles hindering the development of tools	• Limited data availability and/or quality	• A big problem in developing tools is that data availability. There are very little German annotated Corpora for training natural language pipelines. Most tools are trained on, I don't know, pub med or some other biomedical um Corpora, which is not the same language as used in EHR. So we have a huge gap there. (I1)
		• Data protection issues	• To enable the development of these tools you have to allow data to circulate. And the more data is available for all sorts of analysis, the more risks in terms of privacy, confident charity, data protection you are facing. (I6)
		• Risk of bias in training	• The biggest challenge is the bias which you have. I mean all models are trained on a certain baseline data set and obviously the amount of data and the diversity of data you integrate will largely impact the performance of the model. Gender diversity, social diversity, educational diversity, that all plays an impact on how these models perform at the end. (I5)

	Challenges in the deployment of tools	• Models interoperability	• Another challenge is the comparability between models. So if you, for example, train it in hospital A and then you want to apply it in hospital B that might be different and really have challenges. Beside the language barriers, et cetera, there are sometimes different terms, different ways how you document processes that might also impact the performance of the model. (I5)
		• Limitations of model performance	• use case d: All that modelling and simulation is already quite successful on the efficacy side, but much less on the safety side and safety problems. (I6)<br>• Being actively involved in AI research, I'm just a bit more sceptical because I realize what works and what does not work. Medicine is really complex because humans are very complex and diseases are complex. And this is why I think this will also apply to the field of medical studies, this complex and we really need to invest into this. I also see that there is huge potential behind it, but there needs to be investment. (I5)
		• Risk of misuse	• The risk for me doesn't come from the model itself but from who uses it. (I1)<br>• The tool should not unduly influence the decision of patients whether to participate in the trial or not. When you use Amazon, there is an algorithm that profiles you and shows you products accordingly. For medical research, you can't do that. So you can't use this kind of tools to “sell” the trial. (I3)<br>• use case c: There is a risk of discrimination and misuse of tool for other scopes. For example if you then keep this data bank with this categorization and you combine it with other data banks for creating detailed profiles of the patients. (I3)
	Trust issues in the deployment of tools	• Lack of trust in AI	• I'm not an expert in the field of human research but in my field, I haven't come across any tool that I would employ for legal work without human supervision. So, in the field where I can judge the quality of the results, I wouldn't use anything without human supervision. I haven't seen anything that's trustworthy enough. (I2)<br>• AI deciding whether or not to include a patient in a study may be problematic, especially for studies requiring only few patients. Although equal access is actually a general problem independent from AI. You should be able to explain the decision. (I1)<br>• use case d: if that works more and more and better and better. We will have drugs on the, on the market which have only been given to a very small number of real people. And the question is in, how far will patients trust such a medicine? (I4)
		• Risk of breaking trust	• use case a: During a normal screening process there is a contact between the study team and the potential study participants. If you automate the process, this is not there anymore. Everything is done behind the back of participants. And this is an ethical problem. It breaks the trust, which is important for the informed consent process and decision of the patient whether to participate in the trial or not. (I3)<br>• use case a: If we would in standard way, ask every patient that comes to a hospital or to a practice whether he or she would agree that the patient files would be searched for potential um suitability for a clinical trial, then I think it's absolutely not problematic. Without that consent is difficult if it is outside of this trusted doctor, patient relationship. Especially from people outside a specific department. (I4)<br>• use case b: It can be a problem that you abuse trust of the patient. The tool should not influence the decision of patients whether to participate in the trial or not. (I3)<br>• use case c: I think that the game is not worth the candle, it's too much reputational risk for, I would say insufficient biomedical gain....I mean, social media platforms might contain some signals, but I think the quality of the data is insufficient actually. The number of inferences that you have to make the amount of data that you need to train accurate predictions. (I6)<br>• Imagine that you are a clinical centre that is recruiting for a specific trial on a sexually transmitted disease, which is usually an ethically controversial topic. I mean, how would you feel about being selected for a study based on inferences made on your habits? For example, in the sphere of intimate personal life, I mean, people might feel violated, people might feel exposed. (I6)

	Challenges in the quality control of tools	• Tool validation is use-case specific<br><br>• Missing quality standards	• No, I don't think these tools should be certified. Model performance will just be good enough for one specific use case. I don't think you can standardize this in any way. (I1)<br><br>• Competent authorities are not trusting the data that come out of that because there is no agreed quality standard and no, let's say process to judge on whether this is now a good model or a bad model. And I think that is probably the biggest danger in that whole thing when everybody can get started, that we don't have that assessment option. (I4)<br>• use case b: The question is then what happens with that information that is provided through the chatbot then to the machine. For me, it is really not clear which criteria should be used and can be used to, let's say limit ultimately the choice of the patient to whether they are suitable or not because we have not developed that criteria. I would not be prepared to say that a patient who is sceptical and who is afraid of the clinical trial is less suitable than a patient who is euphoric and said you're wonderful. Furthermore, these criteria are impacted by the disease and the status of the disease and the experience of the disease. (I4)<br>• use case d: A very philosophically interesting problem is if synthetic data have to accurately represent the data they've been trained on, then they're going to represent also the social demographic skill of the of the original data. So what is a good synthetic dataset? One that is faithful to the original and therefore replicates also the limitations of the original or one that compensates for the limitation of the original data set? (I6)
Opportunity or Challenge?	Trade-offs of local solutions	• Local solutions are more safe<br>• Language specificity and model interoperability<br>• Tools customization is essential<br><br>**vs**<br><br>• Negative cost-benefit balance<br><br>• Limited data availability and quality<br><br>• Centralization for transparency	•It would be better to deploy an LLM locally and have the patients use it, as it is safer regulatory-wise. I'm really in favour of having local solutions because the way how we talk or how we write or document our data at Hospital A is different from other hospitals. And this might not be a problem if you just want to have information about the study and you want to kind of generate some language output. But it could be a problem if you rely on very specific details, let's say when it comes to inclusion or exclusion. (I1)<br>• The researcher knows based what the research question is and how the study is designed, et cetera. it really allows you to customize the tool in such a way that the impact of the tool is, is much better. And I think that's the question with many of these tools, how much is the level of customization commercial company can offer you? (I5)<br><br>• If you need to train an AI tool specifically for your study, this could mean a lot of resources to do this.... It's such a highly regulated environment and it's really hard to get something going. So I don't think the benefits outweigh the resources that you need to invest in order to develop it right now. (I1)<br>• A big problem in developing tools is that data availability. There are very little German annotated Corpora for training natural language pipelines. Most tools are trained on, I don't know, pub med or some other biomedical um Corpora, which is not the same language as used in EHR. So we have a huge gap there. We cannot have those data silos that we have right now. We need to centralize it more with the risk obviously of losing a bit of privacy. I think many people in, in Switzerland would be willing to do so. Centralization of data and consent management would in the end lead to more information for the patients. (I1)

	Advantages and limitations of anonymization and implications for consent requirement	•Anonymization and enabling innovation<br><br>**vs**	• Personally, I think we really block innovation too much if we require that patients must be informed that their data is used to train such AI tools. (I4)<br>• I don't think patients should be informed otherwise when they have signed a general consent. (I1)
		•Limitations of anonymization	• You also know, dependent on the amount of information you have for a person, if you ask a person 20 questions, I'm pretty sure you could pin point out who that person is. So I think it's a tricky part with anonymization, especially when data sets get aggregated. This is increasingly difficult we just have to be realistic there. (I5)
		•Transparency and consent requirement	• I'm struggling with my own opinion on this because from a legal point of view and as a as a data protection professional, I think that transparency is vital. So yes, patients should be informed always about the use of their data to develop or train tools. On the other hand, I see the practical implications of this. (I2)<br>• The patients should always be informed about the use of the AI tool, it is critical to build this trust that is necessary later also. I would take data only from patients who signed a general consent. It doesn't matter if the data are coded, anonymized, and so on. Even if it's not a research project under the Human Research Act. It's a matter of respect. (I3)<br>• When patients sign the general consent, what we are discussing here is probably not what they have in mind. I'm just not sure whether the general consent is really adequate for this kind of use of patient data. (I2)<br>• For data that is already collected and already consented for clinical research use doesn't require another layer of consent. But um but at least people could be informed...You have at least to inform them that something like a major new technological shift is enabling now a different use of their data and you can ask permission for that. (I6)
	The good and bad of open sourcing and collaboration between stakeholders	• Exploit strengths of different stakeholders	• I would favour a cooperative approach because I think it needs all sides, it needs academia for the basic principles and the responsible approach, but it also needs the industry to push things and to fund things. (I2)<br>• I think everybody who is capable should be involved. I would not see that there's any difference. I mean, there is certainly industry who has um a commercial interest in developing that. There are certainly academic researchers, who are great minds and who have a framework where they can develop these things and, and try that out. So, I think personally, I would not say there's a focus on who should do it as long. (I5)
		• Exploit available models<br><br>**vs**	• It's just really hard to train LLMs from scratch with usable output. You have very good ones available and if your tool performs less than that, people might not be so happy to use it. You have to rely on a base model that is already there because otherwise you cannot make a valid output or something that works good. (I1)
		• Open sourcing enhances the risk of misuse<br>• Problem of sharing data with third parties	• With open source model there is too high risk of misuse. Let's say the model is deployed locally and not everybody has access to it, but only the person who should be enrolled in a study or so on. It's way different than if you would say this is an open model and every person in Switzerland can chat with it and then we want to extract whether or not they're suitable for a study. (I1)<br>• use case b: If there is a discussion with the chatbot, et cetera, I think that's going to be very difficult because also imagine you have a study and in this study, there is innovation behind it. And that, so how would you then transfer this information to a company that generates such a lot of subsequent legal questions and contracts etc.? That would also delay the process. (I5)
		• Open sourcing hinders development	• For the developer there must be an incentive, it can be a financial incentive or recognition or incentive by the society or by the medical society or by the patient community. If I develop myself something and I'm forced by the state or whatever to make it public, without any sort of recognition, then why should I develop it? I'm not against open source, but not in such a way as to hinder development. (I3)

	Impact on fairness and data diversity	• Increased fairness and data diversity<br><br>**vs**<br><br>• Lack of trust<br>•Risk of bias<br>• Risk of inequities	• AI might generate more equal opportunities. Because if an AI model, for example, can adjust its language and the educational background of the person.... And so therefore, I think by having this adaptability, you could get to recruit more diverse populations. (I5)<br>• You're probably more fair, I think really fair because you treat everybody the same way. AI does not have, you know, like certain biases or sympathies towards a person. (I5)<br>• You reach out population that otherwise will not be contacted normally. You broaden your study population and you make it more real life population. (I3)<br><br>• I think, but that's only speculation, I think it could affect participation. Maybe younger people and people who are more open to technological developments would be more keen to participate in research and older and more sceptical people might be less keen, which then would result in bias for your research. (I2)<br>• use case e: If we target, I don't know, neurologists for interviews, then we might say, well, neurologist, they don't have time, they are unlikely to respond. But these are considerations based on general characteristics of your participating sample. It's not based on individual characteristics. So I think that this is where you might have a trade-off. If you are working on general characteristics, I think it's totally innocent to make these predictions. If you are going deeper into more individual data, maybe can become more problematic. (I6)<br>• use case c: I like social media but it's a completely biased world. I mean, people who use social media are not the general public. You would get a very extreme opinions. (I5)
Recommend ations	Recommen dations for tools developme nt	• Bias awareness<br><br>• Risk assessment and quantification	• use case c: I like social media but it's a completely biased world....The general sentiment on social media, with the exception of LinkedIn is very anti science. Unless you want to specifically address those, um you just need to be aware of what the bias is. (I5)<br><br>• Many tools just focus on performance and not on the risk. What we lack as of now is risk assessment. There are very few methods on how to really assess and quantify the risk. And actually all the decisions around these tools should be risk-based. You could have something similar to bug bounty programs for cybersecurity. So those are hackers that you employ and they can look for vulnerabilities in your systems. And then, if they find something, they report it to you and they don't exploit it in a malicious way. You could do the same for AI. (I1)

	Recommendations for tools deployment	• Importance of implementation context	• When it comes to assessing the use of technology in the medical sphere, from an ethical point of view, it's never just about looking at the features of the technology itself. What we implement in the clinic is not only the technology, it is a social system. Around the technology there are actors that operate and maintain it, there are users and subjects. it's really a complex social ensemble that we implement. (I6)<br>• A system that looks for participants based on data, let's say, as a default is framing the participant more in a passive role. The acquisition of data without involving the data subject of course, frames the position of the data subject more passively. But I have the impression that a good architecture could potentially be empowering. We need to be careful with this kind of judgments because it's not that patients are very active today in clinical research. So we're not moving from a state of affairs today in which patients are co-creators of clinical research to a state in to a state of affairs in the future in which, patients are going to be totally passive. I think that these are two extreme scenarios that are not realistic. We really need to be careful about the assumptions that are embedded in technological tools. There is this passive framing of data subjects. It doesn't need to be there but we have to be careful about that. (I6)<br>• use case b: The participant is going to miss the part of the human component of enrolling in a trial which might also play out negatively in terms of enrolment rates...The patient remains a little bit alone. So the deliberation process happens a little bit too much in isolation or this is at least one of the aspects that should be taken into account. It doesn't need to be fully automated. There should there could still be a human involvement in the actual recruitment of the participants. (I6)<br>• And it is really important including the instructions to the investigator and taking the personality of the investigator into consideration, which is really the difficult thing. (I4)
		• Transparency	• The biggest problem that we have for recruitment is lack of trust on the patient side. And everything that could increase the suspicion um about how things are happening are absolutely counterproductive. So I think it's absolutely mandatory that there is complete transparency and openness about potential use of technology here. (I4)<br>• I think you should definitely tell study participants how you recruited them, from social media posts or from EHR, but I don't think it's necessary to say you did it with an AI. So if you have a list in Excel, you wouldn't tell the patients. (I1)
	Recommendations for quality control	• Human supervision	• I think we should still give the final word to a human being whether this is good or not. AI is going beyond the human capacity of the brainpower. But I think the intuition and the experience of an individual is something that an AI cannot achieve in the same way. So I think it would require a second opinion from a human being. (I4)<br>• AI should be a support system. I'm doubtful that the AI can be trained in a way to answer study specific questions where it's just an experience of a physician. So you, you need to have still a human element to it also for acceptance at the moment. (I5)
		• Tools Monitoring	• We need to have also a possibility to supervise the framework, because you can define everything, but if nobody adheres to it, it's useless. (I4)<br>• There should be at some point, somebody that oversees in general, the use of AI tools in the hospital, that oversees what gets in, that monitors how things are unfolding. Somebody quite high up hierarchical that has o oversight responsibilities at the level of the hospital. (I6)
		• Support and reporting system	• I think every tool should have some sort of support hotline in the background. So if you interact with it, you should be able to raise an issue or to say if something is going or something is odd and you need to be able to report this to a person. (I1)

	Overall	• Regulatory framework	• If you look in the internet there's a lot of guidance documents or regulations. But mostly on use cases and application, there is very little on the research. There are very few guidance documents on research. (I3)<br>• I think that is what the regulation should really ensure that there is um a well understandable, clearly defined um definition, a clearly defined scope of things that are generally acceptable. (I4)<br>• I think that some things should be regulated by the law, but arguably a software that recruits participants in a clinical study is not going to be classified as a medical device. But this is not a good reason to say that it should go completely unaccounted for. I mean, somebody should look at how these things work and I think it should be a figure in the hospital of the future. (I6)<br>•Clear rules are required but you should be able to use it without restrictions. It's like you write an article and you use word and nobody is going to ask whether you use word or some other tools. (I1)
		• Agreed quality standards<br>• No certification	• If you require certification, that would massively increase the cost and delay everything. I'm just a bit sceptical by having this extra layer of control and I'm also not aware of a body which could assess such a software. I rather think for this kind of application, there should maybe be a sort of a criteria list for good AI practice or something like you have GCP. (I5)<br>• Some forms of classification might emerge sort of spontaneously from the community. So you know that in clinical research there are reporting standards, for example. These are not imposed by anybody. These are standards because journals use them and researchers accept them. I don't believe that we're going to see a lot of certification, but we might still see some forms of standardization that is likely to emerge spontaneously. (I6)<br>• We have now in the legislation and the clinical trial regulation a requirement to explain the recruitment process in detail to the ethics committee. And I think if there is AI involved, then that would need to be presented in that declaration to the ethics committee. And in this way, then also reviewed by the ethics committee, whether this is acceptable or not. The problem is that ethics committee have also not an agreed judgment, scheme. So to say some are more restrictive than others or more sceptical than others. So we will all we would then also have to work on that and find general rules. (I4)
		• Risk-based approach	• The level of human supervision of these tools also plays a role to decide whether to inform the patients or not about the use of the AI tool in a study. if there's an automated decision without human supervision, then certainly you should inform a patient if it's a decision that affects the patient in any way. (I2)<br>• If training data is anonymized, there's no issue, but a complete anonymization might be an illusion. So maybe there could be a risk based approach. You assess the re-identification risk and then decide how much transparency you need. But it is very difficult to decide on a cut-off point. So that would be the next challenge there. (I2)<br>• The tendency is certainly to become more and more restrictive and demanding here. So I would not be surprised if we would end up in, at some stage, also requiring certification also for these AI tools, especially when there is a medical consequence for the patient. When it is about inclusion of the patient, that would fall for me under an area where I would want to have a control. For recruitment process more in general, I think that would be for me, a grey area where further clarification would be required. (I4)
		• Moral agreement	• This really needs to be put into an ethical framework and that has to be worked out to make sure that it works. In simple words, we need an agreed definition of what is right and what is wrong in this particular area. There is still a long way to go. (I4)
		• Importance of training	• I would rather see a disadvantage regarding attitude of patients toward medical research...This might change with more training of the general public. The perception of AI will massively change. There are some initiatives to educate people about AI, but it's also a selection of people who go there. (I5)<br>• Challenges that I address are mostly privacy based. I think with a careful design of the processes, a careful choice of tools and a careful choice of people using these tools and careful instruction of such people, the challenges can be overcome. (I2)
		• Data protection over entire tool lifecycle	• What many papers never mentioned is that you have more ways to address data protection than just at the training stage. You can have measures implemented also in the fine-tuning of the model or in the deployment of the model. (I1)
