# Supplementary material for: Natural Language Processing in Clinical Research Recruitment: A Scoping Review Enriched with Stakeholder Insights
Source: Ethics Hum Res. 2025 Sep 27;47(5):13–23. doi: 10.1002/eahr.60014 (PMC12476210; doi:10.1002/eahr.60014)
Supplement: Supplementary file 3 — Supporting information [file EAHR-47-13-s003.pdf]

Natural Language Processing in Clinical Research Recruitment: A Scoping Review Enriched with Stakeholder Insights

Lara Bernasconi, Georg Avakyan, Frédérique Hovaguimian, and Regina Grossmann

Figure 1: PRISMA Flow Diagram

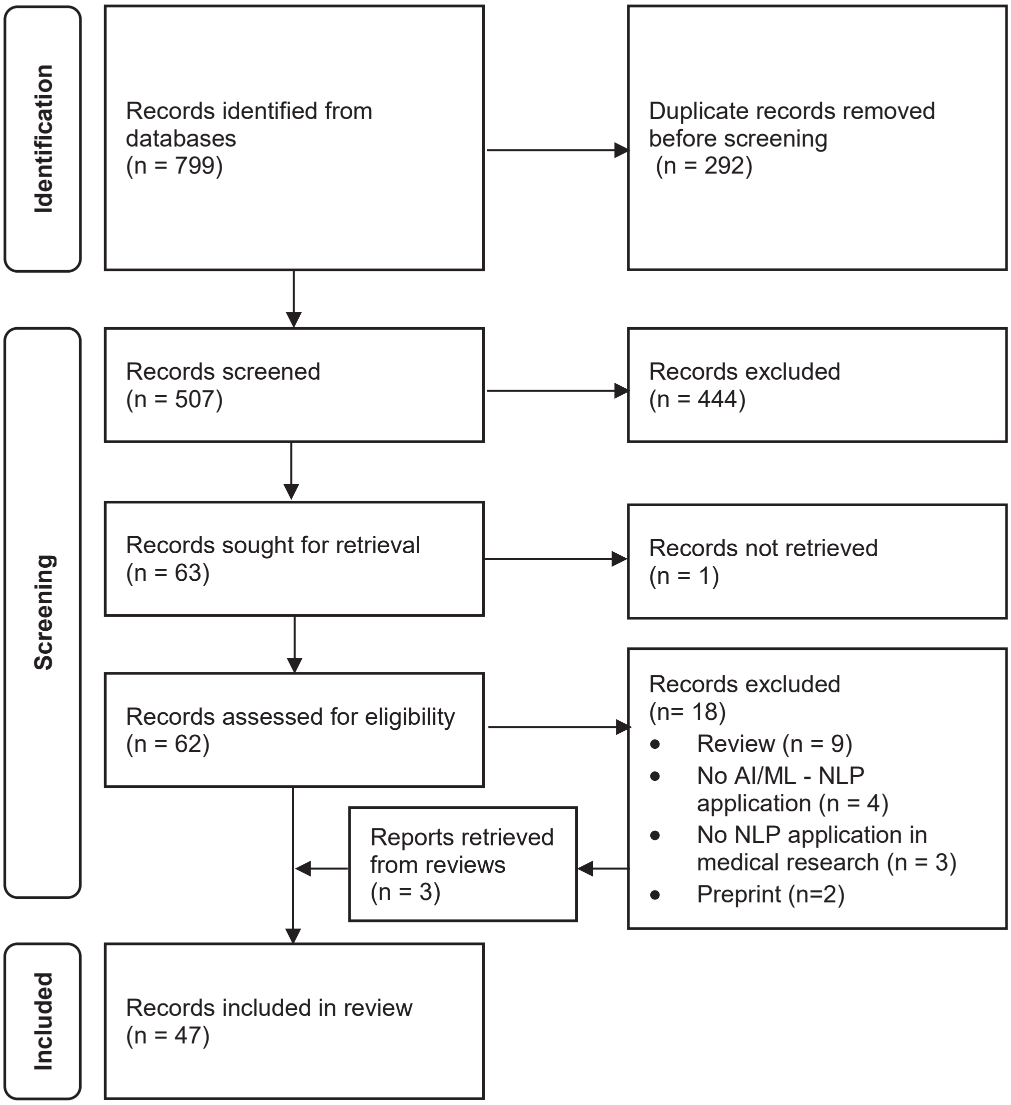

The template diagram has been modified to also reflect the articles sourced from the review papers.
