# Supplementary material for: Natural Language Processing in Clinical Research Recruitment: A Scoping Review Enriched with Stakeholder Insights
Source: Ethics Hum Res. 2025 Sep 27;47(5):13–23. doi: 10.1002/eahr.60014 (PMC12476210; doi:10.1002/eahr.60014)
Supplement: Supplementary file 4 — Supporting information [file EAHR-47-13-s001.pdf]

Natural Language Processing in Clinical Research Recruitment: A Scoping Review Enriched with Stakeholder Insights

Lara Bernasconi, Georg Avakyan, Frédérique Hovaguimian, and Regina Grossmann

Figure 2: Identified NLP Applications to Support Recruitment in Clinical Research (n = 47)

One article has been assigned to both categories "patient identification" and "creation of cohort queries."

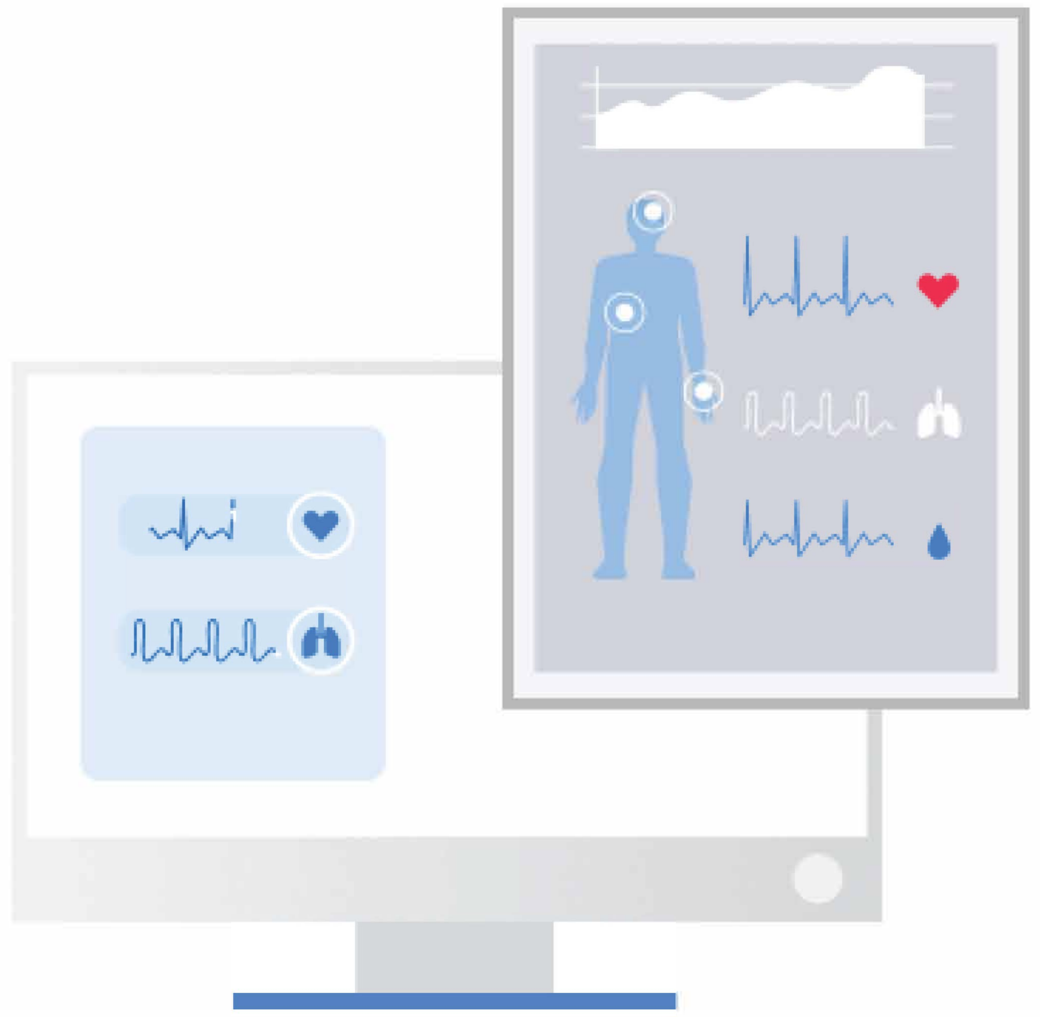

Screening from EHR

Patient identification: **20**

Bi-directional matching: **4**

Patient-centric trial recommendation: **3**

Prediction of screening success: **1**

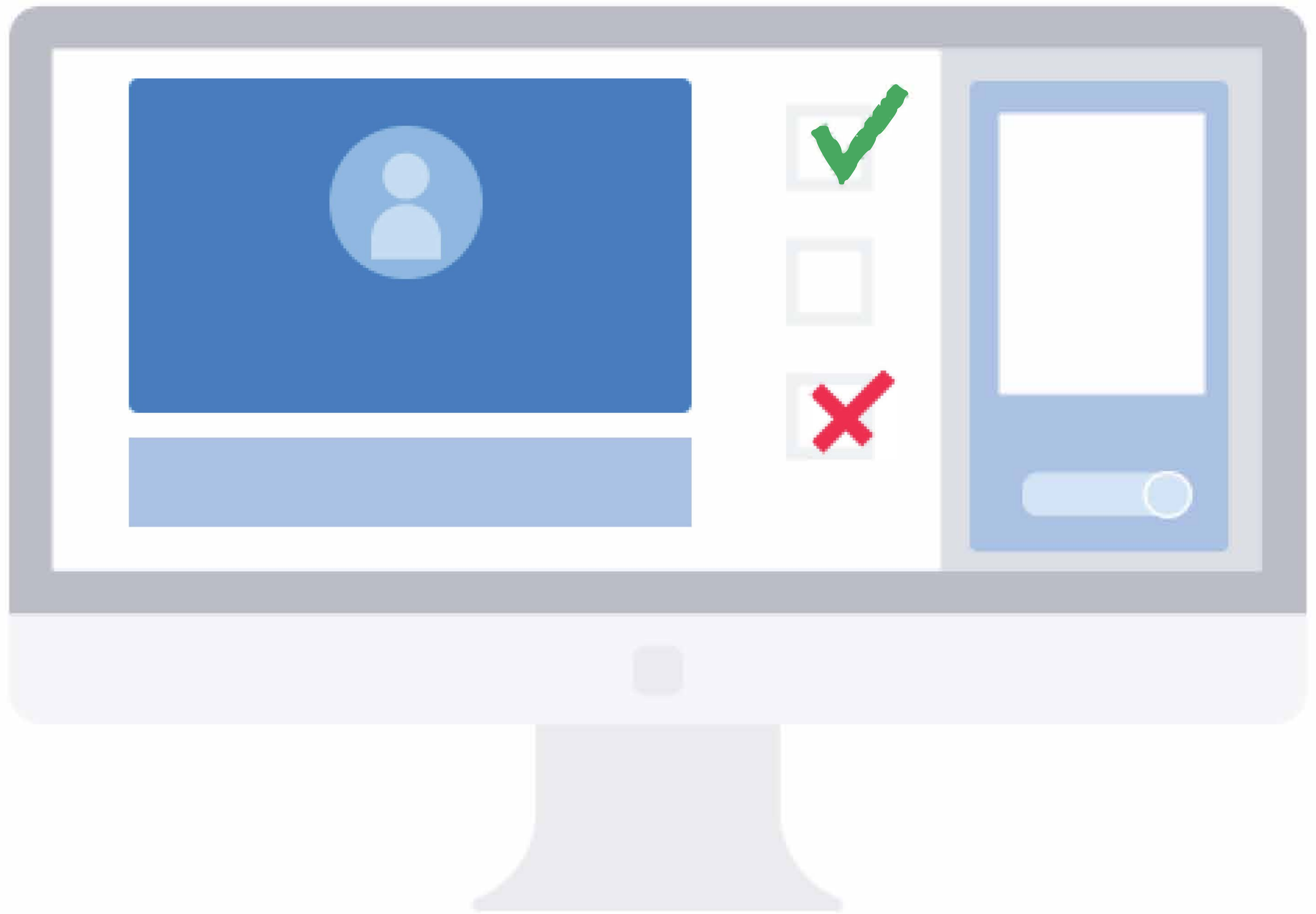

Parsing of Eligibility Criteria

Creation of cohort queries: **8**

Structure/Code eligibility criteria: **5**

Database criteria creation: **1**

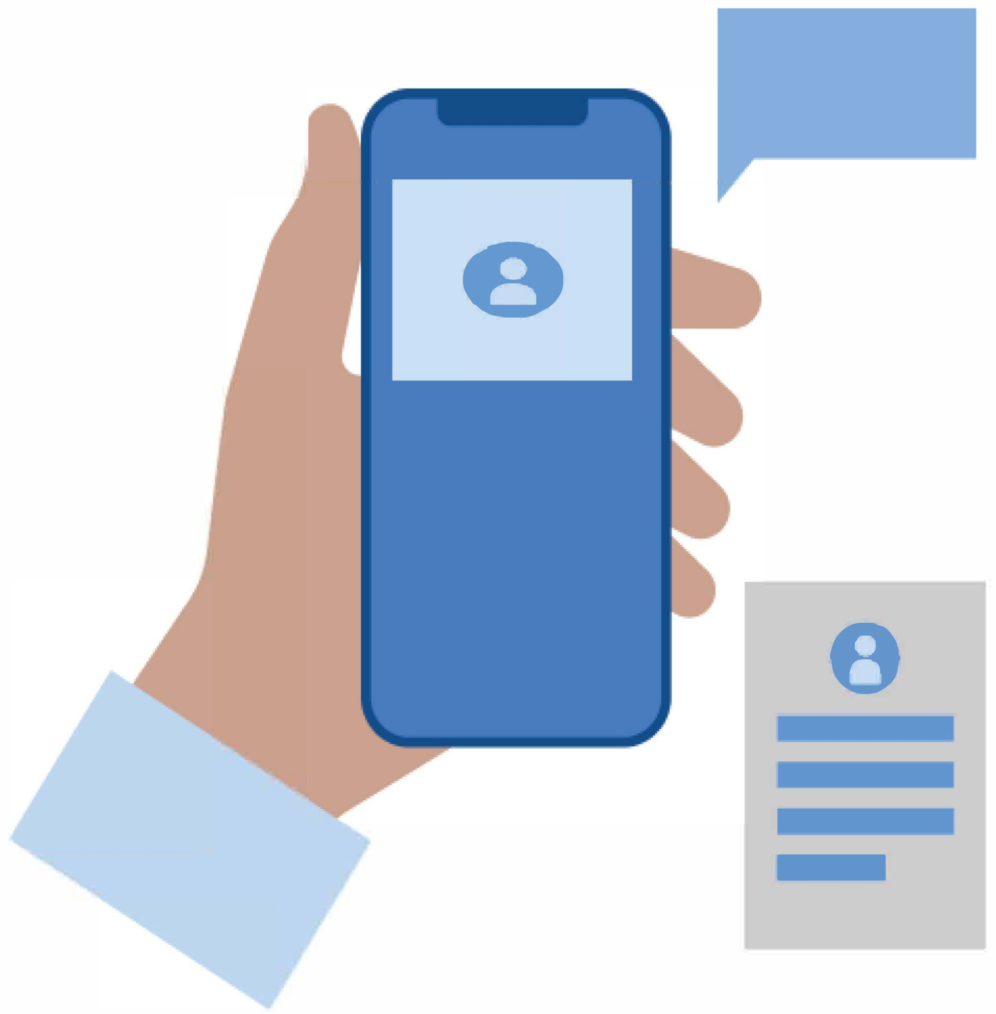

Analysis of Social Media

Sentiment analysis for recruitment enhancement: **1**

User calssification: **1**

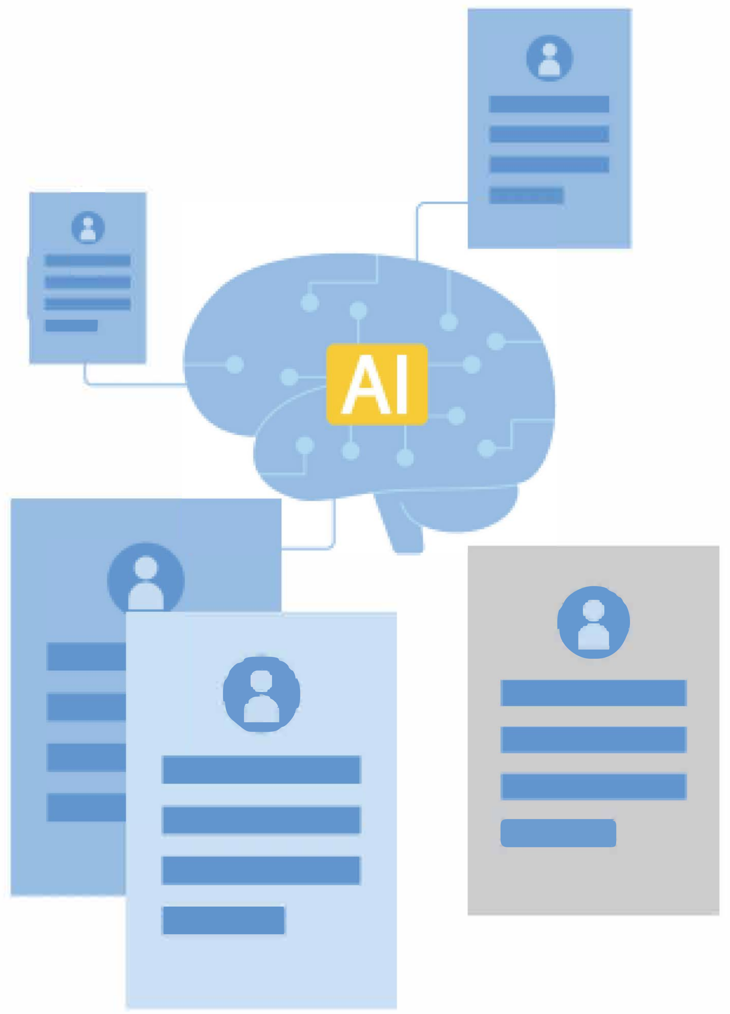

Synthetic Data

Synthetic data: **2**

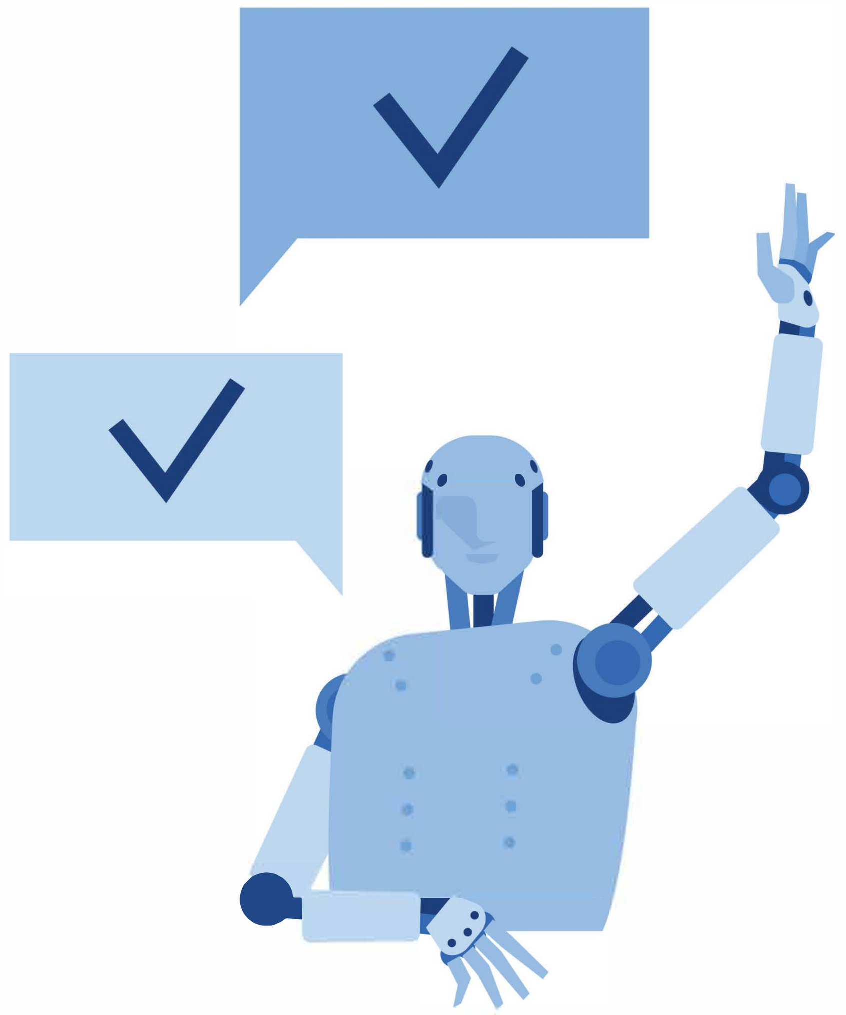

Screening Chatbot

Screening chatbot: **1**

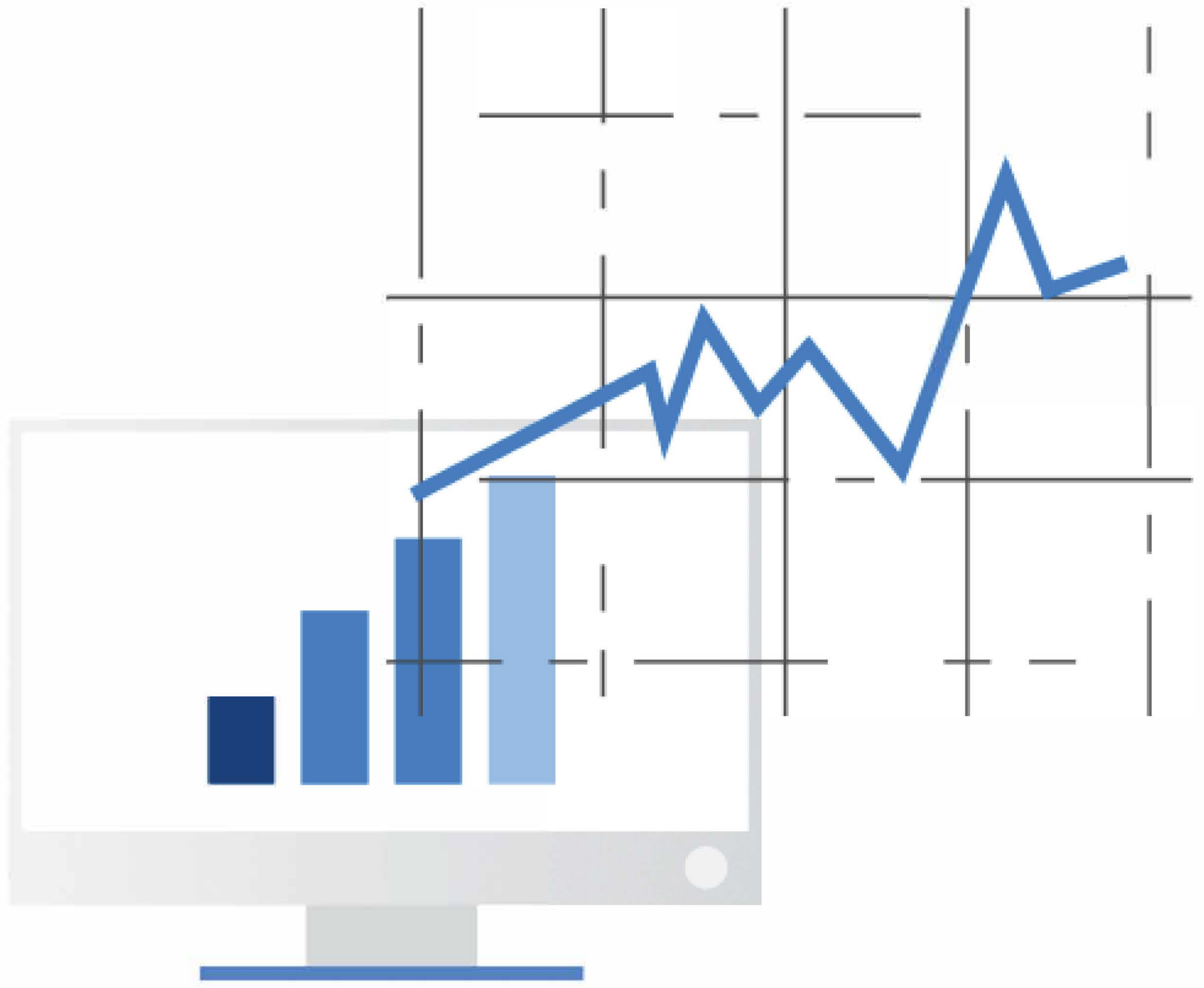

Prediction of Enrolment Rates

Prediction of enrolment rates: **1**
