# Supplementary material for: Natural Language Processing in Clinical Research Recruitment: A Scoping Review Enriched with Stakeholder Insights
Source: Ethics Hum Res. 2025 Sep 27;47(5):13–23. doi: 10.1002/eahr.60014 (PMC12476210; doi:10.1002/eahr.60014)
Supplement: Supplementary file 5 — Supporting information [file EAHR-47-13-s004.pdf]

Natural Language Processing in Clinical Research Recruitment: A Scoping Review Enriched with Stakeholder Insights

Lara Bernasconi, Georg Avakyan, Frédérique Hovaguimian, and Regina Grossmann

Figure 5: Thematic Mind Map

The map illustrates the perspectives of various stakeholders on the use of NLP technologies in clinical research recruitment.

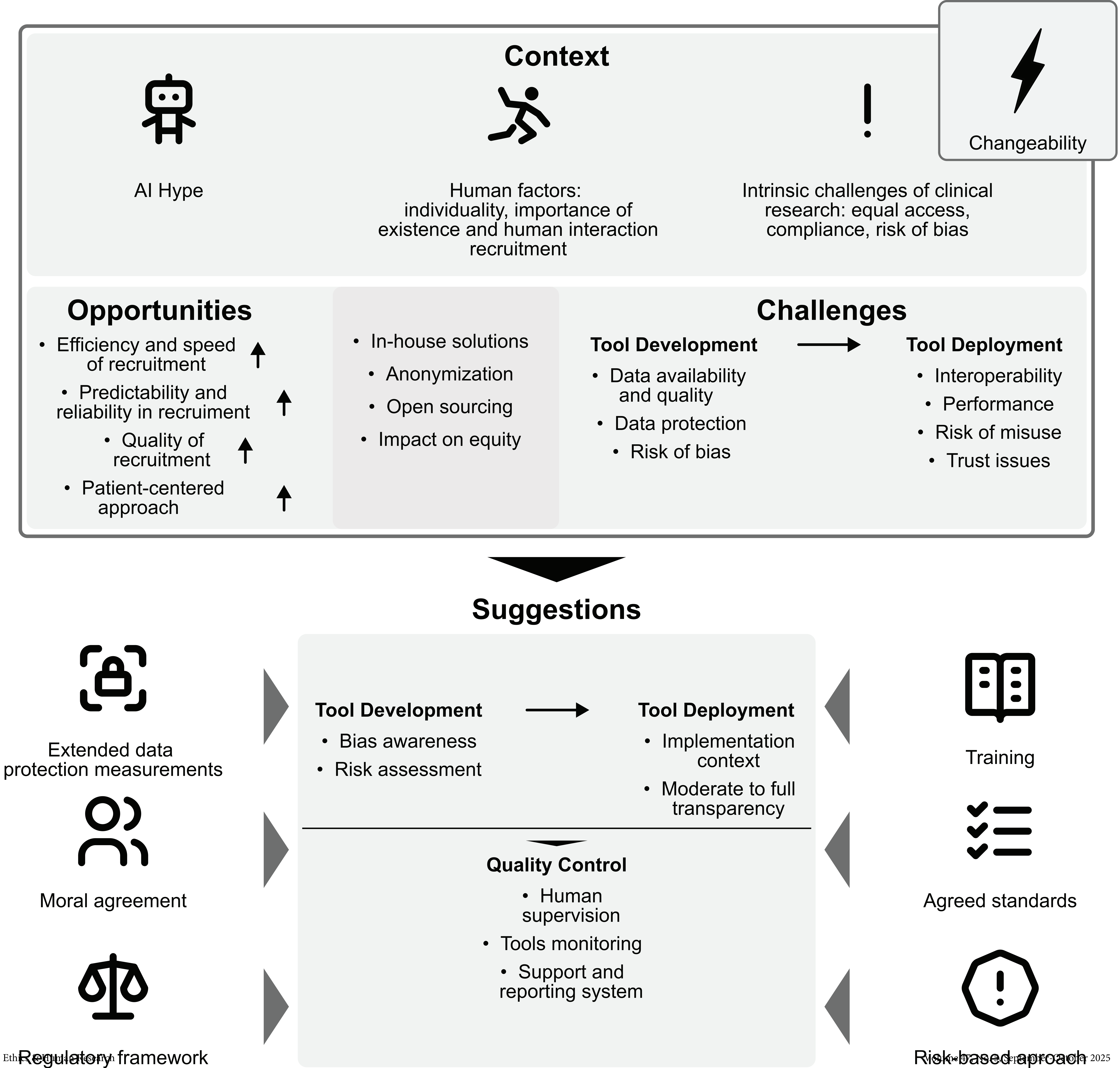
Context
AI Hype
Human factors: individuality, importance of existence and human interaction recruitment
Intrinsic challenges of clinical research: equal access, compliance, risk of bias
Changeability
Opportunities
• Efficiency and speed of recruitment
• Predictability and reliability in recruiment
• Quality of recruitment
• Patient-centered approach
• In-house solutions
• Anonymization
• Open sourcing
• Impact on equity
Challenges
Tool Development
• Data availability and quality
• Data protection
• Risk of bias
Tool Deployment
• Interoperability
• Performance
• Risk of misuse
• Trust issues
Suggestions
Extended data protection measurements
Moral agreement
Regulatory framework
Tool Development
• Bias awareness
• Risk assessment
Tool Deployment
• Implementation context
• Moderate to full transparency
Quality Control
• Human supervision
• Tools monitoring
• Support and reporting system
Training
Agreed standards
Risk-based aproach
